# Cultural Adaptation, Validation and Evaluation of the Psychometric Properties of an Obstetric Violence Scale in the Spanish Context

**DOI:** 10.3390/nursrep13040115

**Published:** 2023-10-03

**Authors:** Héctor González-de la Torre, Paula Nikola González-Artero, Daniel Muñoz de León-Ortega, María Reyes Lancha-de la Cruz, José Verdú-Soriano

**Affiliations:** 1 Department of Nursing, University of Las Palmas de Gran Canaria, Edificio Ciencias de la Salud, C/Blas Cabrera Felipe s/n, CP 35016 Las Palmas de Gran Canaria, Spain; 2 Research Support Unit of Insular Maternal and Child University Hospital Complex of Gran Canaria, Canary Health Service, Avda Marítima del Sur s/n, CP 35016 Las Palmas de Gran Canaria, Spain; 3 Department of Obstetrics and Gynaecology, Insular Maternal and Child University Hospital Complex of Gran Canaria-Canary Health Service, Avda Marítima del Sur s/n, CP 35016 Las Palmas de Gran Canaria, Spain; pgonart@gobiernodecanarias.org (P.N.G.-A.); dmunort@gobiernodecanarias.org (D.M.d.L.-O.); 4 Delivery Room Service, General Hospital of Fuerteventura Virgen de la Peña-Canary Health Service, Carretera del Aeropuerto, Km 1, CP 35600 Puerto del Rosario, Spain; mlancrur@gobiernodecanarias.org; 5 Department of Community Nursing, Preventive Medicine, Public Health and History of Science, Faculty of Health Sciences, University of Alicante (UA), CP 03690 Alicante, Spain; pepe.verdu@ua.es

**Keywords:** obstetric labor, obstetric violence, surveys and questionnaires, validation studies as topic

## Abstract

Obstetric violence refers to dehumanized or derogative treatment of women in their pregnancy, childbirth or postpartum periods and may be manifested in different ways. Currently, there is no tool validated in Spain to measure women’s perception of obstetric violence. The objective of this study was to carry out the cultural adaptation and validation of an existing 14-item obstetric violence scale in the Spanish context and to evaluate its psychometric properties. The research was conducted in two phases: first, a methodological study designed to evaluate content validity, through assessments by eight experts (calculating the Aiken V coefficient) and face validity in a sample of 20 women; second, a cross-sectional study to evaluate construct validity (through confirmatory factor analysis and Rasch analysis), divergent validity against a scale of birth satisfaction, known-groups validity and, finally, reliability. In Phase 1, Aiken V values higher than 0.71 were obtained for all items. Phase 2 was conducted on a sample of 256 women and the fit values for the unidimensional model were RMSEA: 0.070 (95% CI: 0.059–0.105) and GFI: 0.982 (95% CI: 0.823–0.990). The Rasch analysis indicated poor performance of item 2, which was removed. The Omega and Cronbach’s Alpha coefficients were 0.863 and 0.860, respectively. A final 13-item version of the Obstetric Violence Scale was produced, with a total score ranging from 0 (no obstetric violence perception) to 52 (maximum obstetric violence perception). The Obstetric Violence Scale is a reliable and useful tool to measure women’s perception of obstetric violence. This study was not registered.

## 1. Introduction

The choice of an adequate term to designate derogative or inhumane practices and attitudes towards women in their pregnancy, childbirth or postpartum periods is a much debated and controversial issue [1,2]. In English-speaking countries like the United States, such practices are often called “mistreatment at childbirth”, “disrespect” or “abuse” [1,3,4,5]. In other countries, the term “obstetric violence” is preferred [1,6,7].

According to the World Health Organization (WHO), obstetric violence (OV) “is defined as a specific form of violence from health professionals (mainly doctors and nursing staff) towards pregnant women, during childbirth or puerperium. It is a violation of women’s reproductive and sexual rights” [8]. Several forms of obstetric violence are recognized, including five highlighted categories, which are used in legal definitions: routine and unnecessary interventions or medication (on the mother or the infant); verbal abuse or humiliation or physical abuse; lack of suitable equipment or facilities; conducting practices without maternal informed consent (i.e., consent after receiving complete, truthful and sufficient information); and discrimination on cultural, economic, religious or ethnic grounds [8].

OV also includes any practice or attitude affecting a woman’s psychological wellbeing, e.g., treating her as a child, adopting paternalistic or authoritarian attitudes, behaving in a derogatory way, humiliating or insulting her, etc. [1,3,6,7,9].

Both terms, maltreatment/abuse and OV, share the idea of violence based on gender inequality and effects of a patriarchal biomedical system, which usually denies women their autonomy and control over their maternal processes [5,6,10].

OV negatively affects the physical, psychological and emotional health of women who experience it, their relatives and the health professionals that witness it. Excessive, unnecessary or unjustified interventions may often harm women’s health [11]. Performing episiotomy in the absence of fetal risk or without maternal consent is a clear example of this. Episiotomy has been associated with chronic pain and dyspareunia, while it has not been proven to prevent severe perineal trauma [12,13,14]. Therefore, performing routine episiotomy is unjustified and can be considered OV [11,12]. Further examples include obstetric techniques or practices not supported by scientific evidence, like the Kristelle and Hamilton maneuvers, or excessive vaginal examinations [11,15].

Besides physical harm, there are well-documented psychological and emotional aftereffects of OV [15,16]. Victims may experience emotional alterations such as feelings of loneliness or isolation, stress or insecurity [15]. Occasionally, OV causes shame and directly harms women’s self-image and body perception [15]. Such effects may impair women’s sexual and affective dimensions, which makes OV a form of sexual violence [15,17].

Post-traumatic stress disorder is another OV consequence affecting the psycho-emotional dimension. Several studies describe high post-traumatic stress prevalence after childbirth, with rates from 1–6% to 35% [18,19], one of the main risk factors being traumatic experiences during labor due to health providers’ actions or attitudes [20]. This condition also affects women’s partners and children and has an impact on family relationships [21]. Women experiencing verbal or psycho-affective OV are also at higher risk of postpartum depression [22].

OV is a worldwide phenomenon [8,10,17]. Bohren et al. [17] published a review of 65 studies from 34 countries in all continents. Despite considerable heterogeneity, they found that the childbirth experiences of women around the world were often stained with OV. However, the prevalence figures vary largely in different countries [1,23,24,25]. Reported prevalence rates of mistreatment or abuse of women during labor range from 11% in Mexico [26] and 49.4% in Latin America [27] to more than 70% in some African countries [28,29,30]. High OV prevalence rates have also been reported in Europe [1,23,25].

Analysis of the differences in OV prevalence must take into account the complexity of the construct [2,4,31] which, as mentioned, includes several attitudes and practices deeply influenced by social or cultural aspects as well as by medical factors [6,7]. Certain practices considered as OV may be not perceived as such by women or health professionals in certain contexts, which consequently influences the reported rates [2,6,9,32].

The lack of tools specifically validated to measure OV is a further related problem. For example, OV has been evaluated with the Norvold Abuse Questionnaire (Nor-AQ) [33], which is not a specific tool for this type of violence. Recently, a more specific tool has been developed, the Students’ Perceptions of Respectful Maternity Care Scale [34,35], which was validated for some countries [36], although it fails to collect some OV-related aspects.

The PercOV-S Questionnaire (PercOV-S) was designed and validated in Spain for measuring OV as perceived by students of nursing, midwifery and medicine [37]. This tool, which was validated with a sample of 169 students, includes 33 items grouped into two domains: “invisible OV practices” and “visible OV practices”, scored on a Likert-type scale. The PercOV-S shows high reliability and internal validity and has been used in several studies [38,39]. However, all these tools have been designed for healthcare staff and do not evaluate women’s OV perception.

There is a specific scale to evaluate OV as perceived by women, called “Escala de Violencia Obstétrica” (Obstetric Violence Scale), developed by Cárdenas and Salinero [40] based on the “Test de violencia obstétrica” (obstetric violence test) developed by the association “El Parto es Nuestro” [41]. This test was adapted as a scale to measure women’s OV perception based on their memories of certain aspects and situations during labor that are considered related to OV. It is a unidimensional 14-item tool for which some psychometric properties have been evaluated (reliability and construct validity) [40]. Items are scored from 1 (it does not describe what happened to me at all) to 5 (this is definitely what happened to me). The scale was validated with a sample of 367 Chilean women in the Valparaíso region (Chile) [40].

Given the lack of tools to measure OV perception from women’s point of view and since the above-mentioned tool has not been adapted to the Spanish context, the objective of this study was to carry out cultural adaptation and validation of the Obstetric Violence Scale developed by Cárdenas and Salinero [40] in the Spanish context and to evaluate its psychometric properties.

## 2. Materials and Methods

The research was conducted in 2 phases:

Phase 1: content validation through expert judgement, assessment of face validity through a pilot study on a target population and assessment of the understandability of the OV scale;

Phase 2: assessment of construct validity in a sample through factorial analysis and Rasch analysis, divergent validity against a tool that measures satisfaction with labor and assessment of the scale’s reliability (internal validity) and known-groups validity.

### 2.1. Phase 1

In the first phase, a methodological study was conducted to evaluate the content validity and face validity of the Obstetric Violence Scale proposed by Cárdenas and Salinero [40].

#### 2.1.1. Content Validity

Content validity through assessment by a panel of 8 experts from different areas: Experts were selected with the aim of gathering a range of professionals with different views of the studied issue. The experts assessed the pertinence/relevance of every item (whether an item actually evaluated what it was intended to evaluate and the importance of that item in the studied construct). These criteria were expressed in scores from 1 (not pertinent/relevant item) to 4 (very pertinent/relevant item). Based on the experts’ scores, a content validity index was calculated for every item (CVI-i), using the Aiken test with corresponding 95% confidence intervals (95% CI) [42]. This coefficient ranges between 0 and 1 with values closer to 1 indicating better agreement between the judges, which is considered better content validity [42]. Universal-CVI (UA-CVI) was calculated too (proportion of items on an instrument that achieves a relevance rating of 3 or 4 from all the experts).

#### 2.1.2. Face Validity

A pilot test (pre-test) was carried out in a target population to assess the understandability and acceptability of the scale. This pilot test was carried out in a sample of 20 puerperal women, selected by non-probabilistic convenience sampling at the discretion of the research team, provided that they fulfilled the study’s inclusion criteria (older than 18 years; in her first 6 weeks postpartum; last delivery in week 37–42 of pregnancy; live newborn). Participants were asked to quantitatively evaluate the acceptability of items by scoring them from 1 to 4 (1 = poorly acceptable/poorly related to OV; 4 = very acceptable/very related to OV), and the mean acceptability scores were calculated for every item. Additionally, the degree of agreement was calculated for each item as the percentage of participants who scored that item with 3 or 4 over the total number of women. Mean scores over 2 plus agreement scores of over 80% were considered adequate for an item to be acceptable. Participants were also asked to assess the understandability of the scale and to suggest changes if they deemed it appropriate.

Additionally, the final version of the scale was evaluated with INFLESZ [43], a tool validated in the Spanish context used to evaluate text readability and ease of reading by users of healthcare services. It is based on Szigriszt Pazos’ Perspicuity Formula and establishes the following classification system: 0 to 40, very difficult; 40 to 55, moderately difficult; 55 to 65, average difficulty; 65 to 80, fairly easy; and 80 to 100, very easy [43].

### 2.2. Phase 2

#### 2.2.1. Design

A cross-sectional study was designed to obtain a sample for assessing construct validity (through factorial analysis and Rasch analysis), calculation of reliability, evaluation of divergent validity against a scale of satisfaction with labor and known-groups validation.

#### 2.2.2. Study Population

The study population included puerperal women from the province of Las Palmas (Canary Islands, Spain), who visited the office of their primary care midwife in the first 6 weeks postpartum. The inclusion criteria were as follows: older than 18 years, in her first 6 weeks postpartum; last delivery in week 37–42 of pregnancy; live newborn. The exclusion criteria were as follows: inability to read/understand the scale; home birth and/or last delivery by planned caesarean section. Withdrawal criteria were established as follows: incorrect completion of the questionnaire and/or participant’s desire to leave the study.

Eligible participants had given birth at one of the following 4 centers: Center 1 (a public tertiary-level hospital and provincial reference center for high-risk births), Center 2 (a private hospital), Center 3 or Center 4 (regional public hospitals for low-risk pregnancies).

#### 2.2.3. Sampling and Sample Size

Participants were selected through a non-probability convenience sampling procedure. The Cárdenas and Salinero Obstetric Violence Scale included 14 items [40]. According to the classical theory of factor analysis (FA), there must be at least 10–15 subjects per item in the analyzed tool [44,45]. Moreover, since using a polychoric correlation matrix requires a minimum of 200 subjects [44], a minimum sample size of 200 women was estimated for this phase of the study, to allow for confirmatory factor analysis (CFA).

#### 2.2.4. Data Collection Tool and Variables of the Study

A self-administered physical questionnaire was prepared, with a first part collecting several sociodemographic and obstetric variables and a second part containing the Obstetric Violence Scale developed by Cárdenas and Salinero [40].

The variables collected in the first part were age, education level (no education, primary school, secondary school, university), number of deliveries, type of last delivery (eutocic vaginal delivery, dystocic vaginal delivery with forceps, dystocic vaginal delivery with vacuum, unplanned cesarean section), center (Center 1, Insular Maternal and Child University Hospital Complex of Gran Canaria; Center 2, Hospital Vithas Santa Catalina-Las Palmas of Gran Canaria; Center 3 General Hospital of Fuerteventura Virgen de la Peña; Center 4, University Hospital Dr. José Molina Orosa-Lanzarote), episiotomy (Yes/No), consent to perform episiotomy (Yes/No), artificial rupture of membranes (Yes/No), consent to artificial rupture of membranes (Yes/No), labor induction (Yes/No), consent to labor induction (Yes/No), prohibition of receiving food or drink during labor (Yes/No), epidural analgesia (Yes/No), presentation of a childbirth plan (Yes/No) and observation of the childbirth plan (Yes/No).

The second part of the questionnaire included the Obstetric Violence Scale, a tool designed to assess the occurrence of certain OV-related aspects based on postpartum women’s reported memories of their childbirth experience. The 14 items of the scale were scored in a Likert-type format from 1 = “It does not describe what happened to me at all” to 5 = “This definitely happened to me”.

Additionally, to assess divergent validity, 20 women were provided with a version of the questionnaire that included a third part, consisting of a tool to measure maternal satisfaction with labor, namely the Spanish version of the Childbirth Experience Questionnaire (CEQ-E) [46]. The CEQ-E, composed of 22 items grouped into 4 domains, has been reported to reach an overall Cronbach’s alpha coefficient of 0.88 and has been validated and used in the Spanish context [46,47].

#### 2.2.5. Data Collection

Data were collected from 1 December 2022 to 31 March 2023. Participants were recruited through collaborating midwives in 21 primary care centers of the province of Las Palmas (Canary Islands, Spain). All women attending their first to third postpartum control visit, who met the inclusion criteria, were willing to participate and accepted to take part in the study were offered participation. Once they completed the questionnaires, they handled them to their midwife, who kept them until the end of the data collection process.

#### 2.2.6. Data Analysis and Interpretation

Data analysis in Phase 2 was carried out in the following steps:

- Descriptive analysis of the sample and the items: A descriptive analysis of the studied variables and the involved items was conducted. Qualitative variables were expressed in percentages and frequencies; quantitative variables were expressed in measures of central tendency (mean) and dispersion (standard deviation; minimum–maximum values). Symmetry and kurtosis were calculated for each item on the scale;
- Construct validity by factor analysis: A CFA was carried out based on the unidimensional model proposed by Cárdenas and Salinero [40]. The suitability of data for a FA was assessed with the Kaiser–Meyer–Olkin index (KMO) and Bartlett’s statistic. KMO values higher than 0.75 were considered adequate and *p* values ≤ 0.05 were considered statistically significant for Bartlett’s statistic [44].

The suitability of the factorial solution was evaluated through the following indices: Root Mean Square of Residuals (RMSR), Root Mean Square Error of Approximation (RMSEA), Non-Normed Fit Index (NNFI), Comparative Fit Index (CFI), Goodness-of-Fit Index (GFI) and Adjusted Goodness-of-Fit Index (AGFI). For the RMSR, a value of 0.05 was considered an acceptable fit; for the RMSEA, values below 0.05 were considered a good fit and values between 0.05 and 0.08 were considered a reasonable fit [44]. NNFI and CFI values of 0.95 or higher and GFI and AGFI values higher than 0.90 were considered to indicate a good fit of the model [44].

The items’ symmetry and kurtosis values indicated the suitability of using a polychoric correlation matrix for the CFA. Unweighted least squares factor extraction (ULS) and PROMIN oblique rotation were used [48]. The number of factors to be retained was established through a parallel analysis, and the consistency of the retained factors was calculated; 95% confidence intervals were calculated for the item scores and the model measures.

The GH index was calculated to evaluate the degree to which the items reflected a common factor. This index is used to measure the highest percentage of factor variation that the items can measure and two characteristics of the factor analysis: a) the quality of the items as indicators and b) the expected replicability of the solution in different studies. It ranges between 0 and 1 and approaches 1 with increasing magnitudes of factorial load and/or an increasing number of items. Values higher than 0.80 are considered to indicate a well-defined latent variable, likely to remain stable in different studies, while low H values suggest a poorly defined latent variable, likely to change in different studies [49]. H-Latent assesses how correctly a factor can be identified through the continuous latent response variables that underlie the observed item scores, while H-Observed assesses how correctly it can be identified from the observed item scores [49].

To evaluate unidimensionality, the Unidimensional Congruence (UniCo), Explained Common Variance (ECV) and Mean of Item Residual Absolute Loadings (MIREAL) indices were used [50]. UniCo values higher than 0.95, ECV values higher than 0.85 and MIREAL values lower than 0.30 indicated that the data could be considered as essentially unidimensional [50].

- Construct structural validity by Rasch analysis: After verifying that the model was unidimensional, a Rasch analysis was performed. To that end, the OV scores were adapted from a 1–5 range to a 0–4 range.

To estimate the parameters, the Joint Maximum Likelihood estimation (JMLE) method was used for Andrich’s Rating Scale Model. Items’ fit and people’s fit were estimated through the outfit Unweighted Mean Square fit statistic (UMS) and the infit Weighted Mean Square Fit statistic (WMS). Fit index values between 0.8 and 1.2 were considered to be of good fit, while values between 0.5 and 1.5 were considered acceptable fit [51].

To establish the quality statistics, the reliability and separation indices were calculated, both for items and people. For people, reliability values higher than 0.8 and separation values higher than 2 are desirable. Item reliability refers to the degree to which items’ difficulty can be ordered. Item separation provides information on the quality of item location in the latent variable. The assumption of local independence of items was tested with Yen’s Q3 test [52], which evaluates the correlation matrix of residuals. A graphical assessment was also carried out by making an item map.

- Reliability: The Omega and Alpha coefficients were calculated, as well as the Bayes expected a posteriori estimation (EAP scores) [50] reliability of the factorial structure;
- Divergent validity: To explore a possible negative correlation between the OV scale and the CEQ-E questionnaire, the Spearman correlation coefficient was used since the normality test (Shapiro–Wilk test) showed non-symmetry for the OV scale scores’ distribution—considering negative correlation for coefficients (r) higher than −0.5. Standardized scores (z-values) were used to compare both tools;
- Final proposed scale and known-groups validation: Once a final structure of the OV scale was reached, an inferential analysis was conducted to explore the association between the different variables and the score, by comparing groups of women likely to have experienced OV according to several aspects described in the literature. Since data were asymmetric, as evidenced by the Kolmogorov–Smirnov test, the non-parametric Mann–Whitney U test was used to compare means between two groups and the Kruskal–Wallis contrast test was used to compare means between more than two groups, followed by a post hoc contrast (Dwass–Steel–Critchlow–Fligne test) to identify the group comparisons that showed the differences; α values ≤ 0.05 were considered statistically significant. The effect size was calculated for each evaluated association using the Hedges’ formula (Hedges’ g) and Kelley’s epsilon squared measure.

The descriptive and inferential analyses were carried out with the statistical package JAMOVI©v.2.3.24. The FA and model reliability evaluation were carried out with the free-access software FACTOR© Release Version 12.02.01 ×64 bits, and the Rasch analysis was conducted with J Metrik© software (Version 2.3).

#### 2.2.7. Ethical Considerations

This research was approved by the Research Ethics Committee (CEIm) of Hospital Dr. Negrín, Las Palmas, registration number 2022-457-1. All women willing to participate were given an information sheet, in which the objectives of the study were explained, an informed consent form that they had to sign and a revocation form. Data collection forms were anonymous, without names or identification data. All databases were blinded.

## 3. Results

### 3.1. Phase 1

#### 3.1.1. Content Validity

The professional profile of the selected experts (7 women, 1 man) can be found in Supplementary Material Table S1. All items received CVI-i values higher than 0.70. Table 1 shows the scores assigned to each item by each of the experts, as well as the CVI-i values and their corresponding 95% CI. The UA-CVI was 0.6.

#### 3.1.2. Face Validity and Pilot Study in a Target Population

All items received mean scores higher than 3 and degrees of agreement higher than 80%. Item number 12 (your childbirth care experience made you feel vulnerable, guilty or insecure in any sense) received the lowest mean score and degree of agreement (3.2 and 80%, respectively). The scores assigned by the 20 participants in the pilot study can be found in Supplementary Material Table S2, together with the mean score and degree of agreement of each item of the scale.

Participants did not report difficulties in understanding any of the items; therefore, item modifications were not introduced. The perspicuity level for the scale was 64.74 points, which corresponds to a text with a normal level of readability, according to the INFLESZ scale.

### 3.2. Phase 2

#### 3.2.1. Descriptive Analysis of the Sample and the Items

A total of 271 questionnaires were collected; however, 15 of them were withdrawn due to poor data completion; thus, the sample was finally composed of 256 women (*n* = 256). Their mean age was 31.66 years (SD = 5.54) (range: 18–46 years); a total of 159 (62.1%) of them were primiparous, and 97 (37.9%) were multiparous (mean number of deliveries: 1.32, SD = 0.80). Regarding the level of education, 61 (23.8%) women had received primary education, 103 (40.2%) had received secondary education and 92 (35.9%) had completed university studies.

Regarding the type of delivery in last childbirth, 199 women (77.7%) had a normal vaginal delivery, 33 women had an unplanned cesarean section (12.9%), 17 (6.6%) had a dystocic vaginal delivery with forceps and 7 (2.7%) had a dystocic delivery with vacuum. Regarding epidural analgesia, 186 women (72.7%) received analgesia while 70 women (27.3%) did not; a total of 64 women (25.0%) were prohibited from taking food or drink during childbirth.

Regarding other obstetric variables, 32 women (12.5%) underwent episiotomy, 83 (32.4%) underwent artificial rupture of membranes and 116 (45.3%) underwent labor induction. Regarding consent, 17 women with episiotomy (53.1%), 65 women with artificial rupture of membranes (78.3%) and 99 women with labor induction (84.6%) were asked for consent before the interventions.

Finally, participants were asked if they had presented a birth plan and, if so, if the plan had been observed. Most women, 182 (71.1%), did not present a birth plan. From the 74 women (28.9%) who did present a plan, 62 (24.2%) reported that it was observed and 12 (4.7%) reported it was not.

Raw scores and response percentages of each item can be found in Supplementary Material Table S3. Table 2 shows a descriptive analysis of the items as well as the symmetry and kurtosis values.

#### 3.2.2. Construct Validity by Factor Analysis

A CFA was performed based on the proposed 14-item unifactorial model. The KMO values and Bartlett’s statistic indicated an adequate fit of the sample (KMO = 0.824, 95%CI: 0.824–0.888; Bartlett = *p* ≤ 0.001). The one-factor solution showed 60.72% explained variance, with the parallel analysis indicating a single-factor solution. Fit values for this model were RMSEA = 0.070 (95%CI: 0.059–0.105), NNFI = 0.985 (95%CI: 0.898–0.990), IFC = 0.987 (95%CI: 0.914–0.991), GFI = 0.982 (95%CI: 0.823–0.990) and AGFI = 0.979 (95%CI: 0.791–0.989). The Root Mean Square of Residuals (RMSR) yielded a value of 0.085 (95%CI: 0.041–0.121). According to Kelley’s criteria for an acceptable model, the expected RMSR value in this model was 0.062.

Table 3 shows the factorial loads after model rotation and associated 95%CIs. All items showed loads over 0.450, although the confidence intervals were very wide, and three items (numbers 2, 7 and 13) showed loads lower than 0.350 in their confidence intervals.

The results of the analysis showed a H-latent value of 0.969 (95%CI: 0.939–1.000) and an observed H value of 0.513 (95%CI: 0.223–0.743) for a single factor. The unidimensionality analysis showed the following results: UniCo = 0.984 (95%CI: 0.957–0.996), ECV = 0.900 (95%CI: 0.850–0.943) and MIREAL = 0.190 (95%CI: 0.140–0.232). These results supported the model unidimensionality for the scale.

#### 3.2.3. Construct Structural Validity by Rasch Analysis

Given that the factor analysis supported a unidimensional solution, a Rasch analysis was additionally performed to complement the construct validity of the scale. This required adapting the scores to a 0–4 range (0 = “It does not describe what happened to me at all”, 1 = “I’m not sure but I believe/feel that this didn’t happen to me”, 2 = “I’m not sure”, 3 = “I’m not sure but I believe/feel that this happened to me”, 4 = “This definitely happened to me.”).

An initial analysis revealed poor performance of item number 2, which indicated the convenience or removing it from the matrix (Supplementary Material Table S4). A new analysis without item 2 was carried out, and the infit WMS and outfit UMS values were obtained (Table 4). Infit WMS values indicated good fit for all items except item numbers 5 and 12, which presented acceptable fit. Outfit UMS values showed acceptable fit for items 5 and 8 and bad fit for items 11 and 12.

**Figure 1:**
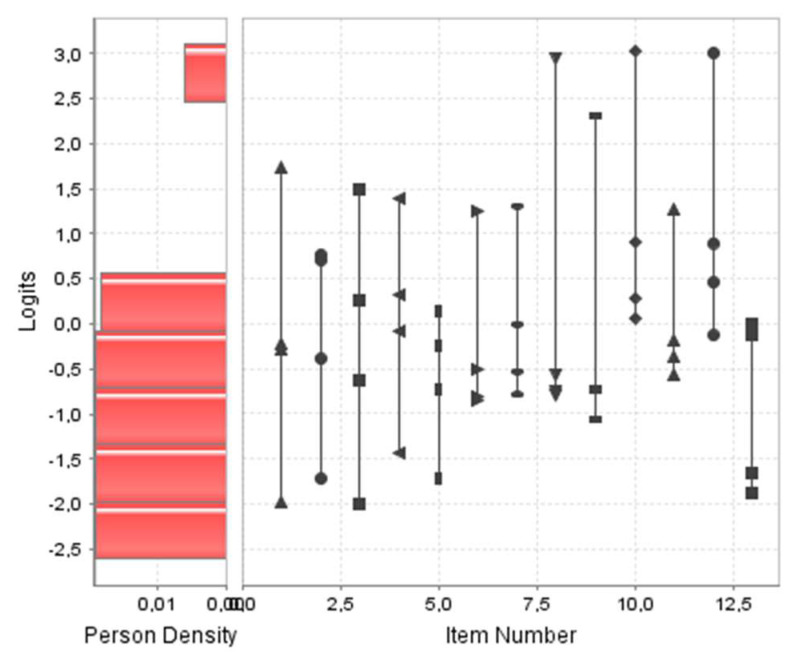
Person—item map of Violence Obstetric Scale.

Figure 1 shows a person–item map of the scale. Regarding the quality statistics, the reliability values of items and people were 0.841 and 0.262, respectively, while the separation index values were 2.300 and 0.596, respectively, which indicated acceptable reliability for the items but not for the people. Regarding the assumption of local independence (Yen’s Q3 test), most values in the correlation matrix were below 0.2–0.3, so the local independence of the items was considered valid (Supplementary Material Table S5).

#### 3.2.4. Reliability

Based on the FA, the EAP was 0.969, with a Factor Determinacy Index of 0.984; the Omega and Cronbach’s Alpha coefficients were 0.863 (95%CI: 0.839–0.888) and 0.860 (95%CI: 0.834–0.883), respectively.

#### 3.2.5. Divergent Validity

**Figure 2:**
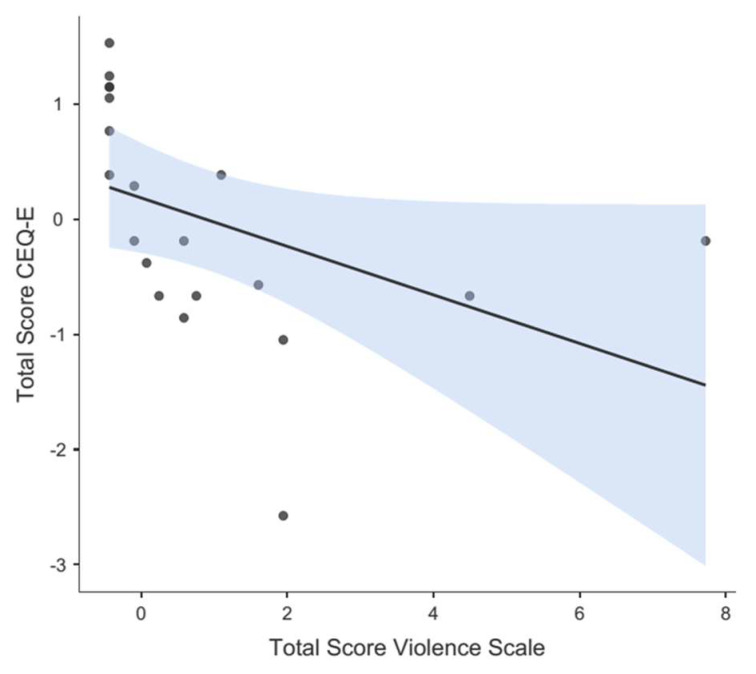
Divergence graph between CEQ—E scores and the Violence Obstetric Scale scores (confidence intervals in blue area).

The mean total score of the CEQ-E questionnaire—completed by 20 women—was 2.91 (SD = 0.47). Spearman’s correlation coefficient was r = −0.794 (*p* < 0.001), evidencing a negative correlation between the total score of the OV scale and the total score of the CEQ-E questionnaire. The correlation is illustrated in the scatter plot in Figure 2 (the higher the OV scores, the lower the satisfaction scores).

#### 3.2.6. Final Proposed Scale and Known-Groups Validation

A final version of OV scale was proposed, with 13 items (item 2 from the original scale was removed) to be scored between 0 and 4, where 0 = “It does not describe what happened to me at all”, 1 = “I’m not sure but I believe/feel that this did not happen to me”, 2 = “I’m not sure”, 3 = “I’m not sure but I think/feel that this did happen to me” and 4 = “This definitely happened to me”. Since the total OV score is calculated by adding individual item scores, it ranges from 0 (no OV perception) to 52 (maximum OV perception). The mean score recorded in the sample for the final version of the scale was 3.03 (SD = 6.55; minimum score = 0; maximum score = 52). The final scale obtained in the Spanish language can be found in Supplementary Material Table S6.

For known-groups validation, the association between certain variables and the scale’s score was analyzed and the effect size was measured for each inference. Statistically significant differences were found for all associations (with varying effect-size magnitudes), except for the association “undergoing labor induction” and “presenting a birth plan”, which showed no differences related to the OV scores. Table 5 shows all the inferences for the bivariate variables.

Finally, in the comparison of means with more than two groups (using the Kruskal–Wallis contrast test followed by a post hoc contrast), no statistically significant differences were found for the education level or the center. However, differences were significant for the type of delivery, with the differences occurring between normal vaginal delivery versus unplanned cesarean section, in the post hoc tests (*p* = 0.001) (Supplementary Material Table S7).

## 4. Discussion

Although OV is an increasingly debated topic [6,53,54], there is currently no adequately validated tool to specifically measure women’s perception of OV. With the aim of solving this problem in our context, this study was focused on the validation of a perceived OV measurement tool, evaluating as many as possible psychometric properties with an as-robust-as-possible methodological approach. We chose to use an already developed tool and to improve certain methodological aspects of the original study by Cardenas and Salinero [40].

In Phase 1, the face validity evaluation, most items were well accepted and no one was considered difficult to understand. Additionally, content validity was analyzed by a group of eight experts from different related disciplines (obstetricians, midwives and nurses). Aiken’s V coefficients above 0.75 were obtained for all items except number 2, with a value of 0.71 (although lower values were found for some items in their confidence intervals).

The interpretation of Aiken’s V is not free of controversy [55]. While some authors consider that values over 0.5 are acceptable, most of them only accept values over 0.70 [56]. Given that confidence intervals are very sensitive to the sample size [55], recruiting more experts may be a solution. Even so, the recorded values provided useful information for making certain decisions in the tool validation process.

The fit values recorded for the model indicated an acceptable fit. Cardenas and Salinero reported RMSEA, the Tucker Lewis Index (TLI; also known as Non-Normed Fit Index (NNFI)) and CFI, but not GFI, in their study [40]. Today, using indices that evaluate different aspects and avoid reporting redundant information is recommended [44]. Ferrando et al. proposed that indices should be communicated based on three criteria: (a) fit of the solution per se (for example, the Goodness-of-Fit Index GFI), (b) comparative fit of the proposed solution against the null model of independence (Non-Normed Fit Index (NNFI) or Comparative Fit Index (CFI)) and (c) relative fit of the model according to its complexity (Root Mean Square Error of Approximation (RMSEA)) [44].

In addition, it is currently recommended to report the RMSR value independently of the model and the estimated solution [44], which was not reported by Cárdenas and Salinero [40]. This value allows one to calculate the Kelley criterion, where the RMSR value is compared with the standard error for zero correlation in the population, thus allowing one to evaluate the suitability of the factorial solution (since if the RMSR is much higher than the expected value, the model should not be considered good) [44,57].

A further aspect to be discussed in the FA of Cárdenas and Salinero [40] is that the authors did not report whether they used linear or nonlinear approximation, which affects the decision of using Pearson’s or a polychoric correlation matrix [44,45,48,58]. Given the marked asymmetry and kurtosis of the scores recorded for the item scores in our questionnaire (higher than those reported by Cardenas and Salinero [40]), it was clear that a polychoric matrix should be used, which entails a more complex model [44,45,48,58].

Furthermore, Cárdenas and Salinero [40] did not indicate whether they performed tests of sample adequacy to a FA, for example, by using the KMO or Bartlett’s statistic [45].

Something that has been verified is the unidimensionality of the scale, from the values of the three used indices. This aspect could be analyzed with a Rasch approach. The Rasch approach is based on classical item response theory (IRT) and allows one to assess a tool from two perspectives: the inherent functioning of the scale and the people who complete it [51,59].

After this analysis, item number 2 (“You were addressed to with nicknames or diminutives, e.g., “mommy”, “chubby”, etc., or treated as if you were unable to understand the processes you were going through”) was removed from the scale. This decision was not only based on the item’s poor performance in the analysis but also on the low scores it received in the content validation by experts. In addition, its low factorial load (less than 0.300) in the confidence interval and its marked asymmetry–kurtosis (also present in the study by Cardenas and Salinero, although to a lesser degree [40]) undoubtedly affected this result. A probable reason for this finding is that some expressions of the item are not used in the context in which this study was carried out. This finding illustrates the importance of the cultural context and peculiarities in the adaptation and validation of tools in different countries, even within the same language [60].

Using a Rasch analysis involves certain assumptions, such as the unidimensionality of the model [51,61] or the existence of minimum scores of 0 for every item. Therefore, the Obstetric Violence Scale scoring system was accordingly adapted to a 0–4 range. Thus, the overall score of the scale ranged from 0, which means no perceived OV, to a maximum of 52, which means maximum perceived OV. In our opinion, this adaptation improves scale interpretation and offers better guidance for the practical use of the tool. Cardenas and Salinero [40] did not establish a clear measurement system nor did they provide measurement cut-off points for the scale.

Finally, the Rasch approach requires checking of the local independence of items, for example, by using Yen’s Q3 test [52]. Traditionally, reference values of 0.2–0.3 have been used; however, there is no homogeneous criterion, since this value depends on the size of the sample, the number of items and the number of responses involved [51,52].

From the usual point of view, the Obstetric Violence Scale’s reliability (internal consistency) analyzed with the Omega and Cronbach’s Alpha coefficients was adequate, with values above 0.80 for both coefficients [62], similar to those reported by Cárdenas and Salinero (0.83 and 0.88, respectively) [40].

In this study, the Rasch analysis allowed us to evaluate the person separation reliability, thus describing people’s separation according to their scoring pattern [61]. Better separation entails more accurate measurement [61]. The higher the value, the better the separation; usually, a minimum value of 2 or higher is used for the index, which indicates that the tool can separate people from at least two strata, for example, low and high capacity [51,61].

The separation indexes lead to the conclusion that reliability was acceptable for the items but not for the people, which suggests that this questionnaire may not be sensitive enough to measure OV in our context. This finding might be due to different reasons: first, a larger sample may be needed; second, OV may be absent or scarcely prevalent in our context; third, women may be unaware or have a little knowledge of OV, which could still be an invisible practice in our health system. These three possibilities should be addressed in future studies.

The psychometric assessment of the tool suggests that, in contexts with low OV levels, its usefulness for the fine measurement of OV is limited. However, the evaluation of other psychometric properties indicates that it may be actually useful in detecting OV. Thus, the evaluation of divergent validity against a scale of satisfaction with labor confirmed that the greater the satisfaction, the lower the OV perception, which is consistent with the theoretical model.

The results of the known-groups validation were also clear. An inferential analysis showed that women undergoing certain interventions perceived more OV, especially if they had not given consent, with statistically significant differences and considerable effect sizes (consent to episiotomy, consent to artificial rupture of membranes and consent to induction of labor). These findings are in line with other studies conducted in our context [11,63] and with the results of Cardenas and Salinero [40].

In this regard, it is important to point out that informed consent (regulated in Spain by Law 41/2002 14 November 2002 on patients’ autonomy and rights and duties regarding information and clinical documentation [64]) must be requested from all patients before any intervention and not doing so entails OV [4,6,8,9].

A further related aspect concerns the presentation of a birth plan. It was evident that this tool is still underused by women in our context (only 28.9% of the total sample presented a birth plan). It was found that women that presented a birth plan that was not observed perceived more OV, with statistically significant differences and a considerable effect size. This might be due to the fact that those women had more information on the delivery and postpartum processes and were, thus, able to identify OV-related situations or procedures, especially those considered invisible practices [1,6,31,37].

The use of birth plans is controversial. Some studies indicate that it is associated with women’s greater dissatisfaction due to unfulfilled expectations during labor [65,66]. However, the problem may lie in the lack of effective communication between healthcare users and providers. Birth plans may serve as a vehicle for such communication and improve women’s satisfaction and feeling of control over the process [67]. The results support their potential importance in the management of OV-related situations.

While in the study by Cárdenas and Salinero, the highest score was assigned to item 5 (“It was difficult or impossible for you to ask questions or express your fears or concerns because nobody answered, or they answered in a bad way”) [40], in this study, it was assigned to item 14 (“During or after labor you felt exposed to the gaze of other people unknown to you (exposure to strangers)”). In the PercOV-S scale, failure to preserve women’s privacy during childbirth is included in the invisible practices domain of OV [37]; therefore, it could remain unnoticed by health professionals, though not by women, as found in this study and described in other ones [38]. This aspect should be taken into account and policies should be applied to warrant women’s privacy during childbirth, especially in centers with educational activities and high turnover of trainees. In the context of OV, intimacy has not been given proper attention as compared to other practices [68,69]; however, it is one of the reasons why many women seek certain settings to give birth in, e.g., their homes [70].

A study by Mena de Tudela et al. showed that private healthcare centers were more prone to invasive practices, something directly related to higher perceived OV [11]. However, no significant differences in OV perception were found in our study, neither between public or private centers, nor between centers with different levels of care. These results should be interpreted with caution though, due to the reduced sample size.

No statistically significant perceived OV differences were found in connection with education level. However, there was a tendency toward higher perceived OV in women with higher levels of education (primary studies M = 1.48, secondary studies M = 3.06, university studies M = 4.03). This finding supports the hypothesis that information is a key factor in women’s awareness and perception of OV, although no studies on such a relationship have been found in the literature.

Results evidence that some women claim having suffered OV to different degrees, both through practices or techniques applied without prior consent and through disrespectful treatment from health professionals. Having a reliable tool to quantify such phenomena can help detect and alleviate this problem. Further studies are required to propose cut-off points for this scale. However, any score other than zero should be worrying and should require intervention.

Regarding the limitations of the study, several points should be highlighted. First, this scale has been used to date only in Spanish-speaking contexts, so its use in other countries is subject to corresponding cross-cultural adaptations and the results obtained in the present study should not be generalized to other settings. Since the temporal reliability of the tool was not assessed, it cannot be ruled out that women’s OV perception is a construct affected by time. Furthermore, analyzing the convergent validity against a different tool could also be of interest. Although there are few tools used to specifically assess OV perception, convergent validity could be evaluated against the PercOV-S to investigate similarities or differences between the perceptions of users and health professionals.

Finally, further studies with different populations are required, since the scale’s reliability was acceptable for the items but not for the women in the sample. It should be taken into account that the studied construct is rather complex [2,3,4,5] and involves both women’s internal factors (perceptions according to education, knowledge, previous experiences and feelings) and external factors (provided healthcare, which may vary in different contexts, centers, regions, countries and/or involved health professionals). New studies with larger samples and women from other regions are needed to verify the performance of the scale in settings with different OV levels.

## 5. Conclusions

OV is a frequent problem with serious physical and psychological consequences for women. Despite being a topic of much debate in recent years, there are no specific validated tools to assess women’s perception of OV. The results of the present study indicate that there are women who perceive different degrees of OV during childbirth, especially when certain interventions are performed without their consent. The Obstetric Violence Scale is a tool with adequate psychometric properties, reliable and useful for measuring women’s perception of OV in Spanish-speaking countries, despite some limitations, especially in low-OV contexts. It is also easy to use by women. Tools with higher sensitivity are needed to evaluate and measure all OV-related aspects. The design of these instruments should take into account cultural peculiarities and the context in which they will be used.

## Figures and Tables

**Table 1:** Scores and content validity index for each item obtained by Aiken’s test.

	Expert 1	Expert 2	Expert 3	Expert 4	Expert 5	Expert 6	Expert 7	Expert 8	CVI-i [95%CI] *
Item 1	4	4	4	4	3	4	4	3	0.92 [0.74–0.98]
Item 2	3	4	4	2	3	4	3	2	0.71 [0.51–0.85]
Item 3	3	4	4	1	3	4	4	4	0.79 [0.60–0.91]
Item 4	3	4	4	2	4	4	4	3	0.83 [0.64–0.93]
Item 5	4	4	4	2	4	4	4	3	0.88 [0.69–0.96]
Item 6	4	4	4	4	4	4	4	4	1.00 [0.86–1.00]
Item 7	3	4	4	3	3	4	3	3	0.79 [0.60–0.91]
Item 8	4	4	4	3	3	4	3	3	0.83 [0.74–0.93]
Item 9	4	4	4	3	3	4	4	3	0.88 [0.69–0.96]
Item 10	4	4	4	3	3	4	3	3	0.83 [0.64–0.93]
Item 11	4	4	4	3	4	4	4	3	0.92 [0.74–0.98]
Item 12	3	4	4	4	3	3	3	2	0.75 [0.55–0.88]
Item 13	4	4	4	3	2	3	3	3	0.75 [0.55–0.88]
Item 14	4	4	4	3	3	4	4	3	0.88 [0.69–0.96]

* Content Validity Index item (Confidence intervals at 95%).

**Table 2:** Descriptive analysis of the items in the Obstetric Violence Scale.

Item	M [95%CI] *	SD **	Symmetry ***	Kurtosis ***
1—Members of the healthcare staff made ironic or derogative comments or made jokes about your behavior.	1.22 [1.09–1.36]	0.85	3.90	13.91
2—You were addressed to with nicknames or diminutives (e.g., mummy, chubby, etc.) or treated as if you were unable to understand the processes you were going through.	1.10 [1.02–1.19]	0.55	5.90	35.06
3—You felt treated as a child or neglected by the staff, as if you were unable to make decisions about what was happening to you before, during or after delivery.	1.25 [1.13–1.39]	0.82	3.55	12.11
4—You were somehow criticized for expressing your emotions (cry, scream of pain, etc.) during labor or delivery.	1.24 [1.10–1.38]	0.86	3.70	12.54
5—It was impossible for you to ask queries or express your fears or concerns because nobody answered, or they answered in a bad way.	1.18 [1.07–1.31]	0.74	4.34	18.20
6—You were subjected to medical procedures without being asked for your consent or without explanation as to why such procedures were needed.	1.37 [1.21–1.54]	1.03	2.79	6.47
7—At the moment of delivery, you were compelled to keep lying on your back despite expressing your discomfort with that position.	1.27 [1.13–1.42]	0.90	3.32	9.83
8—You were compelled to stay in bed and prevented from walking or seeking the position you needed.	1.21 [1.09–1.35]	0.79	3.88	14.26
9—You were not allowed to be accompanied by someone you trusted.	1.15 [1.04–1.27]	0.71	4.75	21.46
10—You were prevented from having immediate contact with your newborn, before the doctor took him/her away (caressing, holding him/her in your arms, etc.).	1.21 [1.09–1.35]	0.83	3.83	13.36
11—After delivery, they made you feel you had not behaved up to what was expected of you (that you had not “helped”).	1.08 [1.00–1.16]	0.48	6.55	44.21
12—Your childbirth care experience made you feel vulnerable, guilty or insecure in any sense.	1.23 [1.11–1.37]	0.80	3.59	12.22
13—After delivery, you were denied the opportunity to use a birth control device or procedure (IUD, tubal ligation, etc.).	1.09 [1.01–1.17]	0.50	6.17	39.37
14—During or after labor, you felt exposed to the gaze of other people unknown to you (exposure to strangers).	1.46 [1.28–1.65]	1.16	2.41	4.24

* Mean (95% confidence interval), ** Standard deviation, *** Polychoric correlation is recommended for univariant item distributions that are asymmetric or with excessive kurtosis. In case both indices are lower than 1, Pearson’s correlation is recommended.

**Table 3:** Unrotated loading matrix with factor loadings of the one-dimensional model for Obstetric Violence Scale.

Items	Factor 1	95%IC *
1—Members of the healthcare staff made ironic or derogative comments or made jokes about your behavior.	0.721	[0.354–0.878]
2—You were addressed to with nicknames or diminutives (e.g., mummy, chubby, etc.) or treated as if you were unable to understand the processes you were going through.	0.645	[0.299–0.892]
3—You felt treated as a child or neglected by the staff, as if you were unable to make decisions about what was happening to you before, during or after delivery.	0.842	[0.594–0.963]
4—You were somehow criticized for expressing your emotions (cry, scream of pain, etc.) during labor or delivery.	0.748	[0.387–0.912]
5—It was impossible for you to ask queries or express your fears or concerns because nobody answered, or they answered in a bad way.	0.884	[0.470–1.000]
6—You were subjected to medical procedures without being asked for your consent or without explanation as to why such procedures were needed.	0.745	[0.364–0.868]
7—At the moment of delivery, you were compelled to keep lying on your back despite expressing your discomfort with that position.	0.486	[0.188–0.739]
8—You were compelled to stay in bed and prevented from walking or seeking the position you needed.	0.790	[0.377–0.918]
9—You were not allowed to be accompanied by someone you trusted.	0.695	[0.334–0.898]
10—You were prevented from having immediate contact with your newborn, before the doctor took him/her away (caressing, holding him/her in your arms, etc.).	0.771	[0.375–0.911]
11—After delivery, they made you feel you had not behaved up to what was expected of you (that you had not “helped”).	0.891	[0.491–1.000]
12—Your childbirth care experience made you feel vulnerable, guilty or insecure in any sense.	0.957	[0.734–1.000]
13—After delivery, you were denied the opportunity to use a birth control device or procedure (IUD, tubal ligation, etc.).	0.473	[−0.489–0.764]
14—During or after labor, you felt exposed to the gaze of other people unknown to you (exposure to strangers).	0.755	[0.460–0.949]

* 95% confidence interval.

**Table 4:** Fit values of the items according to the Joint Maximum Likelihood estimation method in the Rasch analysis.

Item	Difficulty Index *	Infit WMS **	Outfit UMS **
1—Members of the healthcare staff made ironic or derogative comments or made jokes about your behavior.	−0.09	1.09	1.17
3—You felt treated as a child or neglected by the staff, as if you were unable to make decisions about what was happening to you before, during or after delivery.	−0.08	0.85	0.83
4—You were somehow criticized for expressing your emotions (cry, scream of pain, etc.) during labor or delivery.	−0.11	1.18	1.39
5—It was impossible for you to ask queries or express your fears or concerns because nobody answered, or they answered in a bad way.	0.02	0.77	0.58
6—You were subjected to medical procedures without being asked for your consent or without explanation as to why such procedures were needed.	−0.32	1.08	1.06
7—At the moment of delivery, you were compelled to keep lying on your back despite expressing your discomfort with that position.	−0.11	1.06	1.08
8—You were compelled to stay in bed and prevented from walking or seeking the position you needed.	−0.00	0.93	0.76
9—You were not allowed to be accompanied by someone you trusted.	0.10	1.16	1.16
10—You were prevented from having immediate contact with your newborn, before the doctor took him/her away (caressing, holding him/her in your arms, etc.).	−0.03	1.02	0.95
11—After delivery, they made you feel you had not behaved up to what was expected of you (that you had not “helped”).	0.54	0.86	0.37
12—Your childbirth care experience made you feel vulnerable, guilty or insecure in any sense.	0.02	0.58	0.45
13—After delivery, you were denied the opportunity to use a birth control device or procedure (IUD, tubal ligation, etc.).	0.53	1.09	1.13
14—During or after labor, you felt exposed to the gaze of other people unknown to you (exposure to strangers).	−0.46	1.21	1.17

* Difficulty index: Indicates, in this case, the highest values of violence, ** Outfit Unweighted Mean Square fit statistic (UMS) and infit Weighted Mean Square Fit Statistic (WMS); Fit index values between 0.8 and 1.2 meant a good fit and values between 0.5 and 1.5 meant an acceptable fit.

**Table 5:** Mean, standard deviation, *p*-value and effect size for every inference considered in the known-groups validation.

Variables	M (SD) *	*p*-Value **	Effect Size ***
Parity			
Primiparous (n = 159)	3.59 (7.39)	0.040 **	0.23
Multiparous (n = 97)	2.11 (4.75)		
Episiotomy			
No (n = 224)	2.63 (5.97)	0.012 **	0.50
Yes (n = 32)	5.84 (9.32)		
You were asked consent for episiotomy			
No (n = 15)	8.93 (9.87)	0.010 **	0.65
Yes (n = 17)	3.12 (8.13)		
Artificial rupture of membranes (ARM)			
No (n = 173)	2.38 (6.43)	≤0.001 **	0.31
Yes (n = 83)	4.40 (6.62)		
You were asked consent for ARM			
No (n = 18)	9.78 (7.26)	≤0.001 **	1.14
Yes (n = 65)	2.91 (5.63)		
Inducing labor			
No (n = 140)	2.81 (6.80)	0.195	0.07
Yes (n = 116)	3.29 (6.24)		
You were asked consent for inducing labor			
No (n = 18)	6.83 (7.23)	≤0.001 **	0.68
Yes (n = 99)	2.70 (5.85)		
Prohibition of receiving food			
No (n = 192)	2.74 (6.52)	0.018 **	0.18
Yes (n = 64)	3.89 (6.61)		
Epidural analgesia			
No (n = 70)	1.56 (3.84)	0.006 **	0.31
Yes (n = 186)	3.59 (7.24)		
Presentation of a childbirth plan			
No (n = 182)	2.40 (5.75)	0.066	0.34
Yes (n = 74)	4.58 (8.03)		
Your childbirth plan was observed			
No (n = 12)	12.42 (12.93)	≤0.001 **	1.28
Yes (n = 62)	3.04 (5.70)		

* Mean (standard deviation); ** Statistically significant *p* ≤ 0.05 (Mann–Whitney U-test); *** Effect size according to Hedges (Hedges’s g): it considers both groups’ variances and sizes, Values < 0.2 indicate small effects, 0.5 indicates medium effect and 0.8 indicates large effect.

## Data Availability

The data used in this research are confidential and are protected in a coded and anonymized database kept by the research group in accordance with Spanish regulations. However, the raw data from the Violence Obstetric Scale (response to each item) and without the rest of the sociodemographic obstetric variables could be shared with those researchers who contact the corresponding author if requested with a reasoned and logical request.

## Supplementary Materials

The following supporting information can be downloaded at: https://www.mdpi.com/article/10.3390/nursrep13040115/s1, Table S1: Professional profiles of the experts participating in the content validation process; Table S2: Scores from the pilot test with 20 postpartum women, means scores and degree of agreement of each item of the scale; Table S3: Frequencies and response percentages of each item of Obstetric Violence Scale; Table S4: First Rasch analysis by Joint Maximum Likelihood estimation (JMLE) with all the items.; Table S5: Correlation matrix. Yen’s Q3 Test; Table S6: Obstetric Violence Scale (Spanish); Table S7: Means, standard deviations, *p*-value, contrast statistic and effect size for variables in more than two groups.

## Public Involvement Statement

No public involvement in any aspect of this research.

## Guidelines and Standards Statement

This manuscript was drafted against the STROBE for a cross-sectional study, descriptive research (Phase 2) and COSMIN (Consensus-based Standards for the selection of health Measurement In-struments) guidelines for reporting results of measurement instrument studies.
