# Supplementary material for: Cultural Adaptation, Validation and Evaluation of the Psychometric Properties of an Obstetric Violence Scale in the Spanish Context
Source: Nurs Rep. 2023 Oct 3;13(4):1368–87. doi: 10.3390/nursrep13040115 (PMC10594477; doi:10.3390/nursrep13040115)
Supplement: Supplementary file 1 [file nursrep-13-00115-s001.zip › nursrep-2604378-supplementary/Supplementary Material S2 Nursing Reports VO.pdf]

Women	Item 1	Item 2	Item 3	Item 4	Item 5	Item 6	Item 7	Item 8	Item 9	Item 10	Item 11	Item 12	Item 13	Item 14
1	4	4	4	4	4	4	4	4	4	4	4	3	4	4
2	4	4	4	4	4	4	4	4	4	4	4	4	4	4
3	3	4	4	3	4	4	3	3	3	4	3	3	3	3
4	4	4	4	3	3	4	4	4	4	4	4	4	4	3
5	4	4	4	4	4	4	4	4	4	4	4	1	4	4
6	4	4	4	4	3	4	4	4	4	4	4	3	3	2
7	4	4	3	4	4	4	4	4	4	4	4	4	4	4
8	4	3	4	4	4	4	4	4	3	4	4	3	4	4
9	3	1	1	2	3	4	4	3	3	4	3	4	4	4
10	4	4	1	4	4	4	4	4	4	4	4	4	4	4
11	4	4	4	4	4	4	4	3	4	4	4	4	2	3
12	4	4	4	4	4	3	3	3	4	4	4	3	3	3
13	4	4	4	4	4	4	4	4	4	4	4	4	4	4
14	4	4	3	4	1	4	2	3	3	4	3	2	2	4
15	4	3	4	4	4	4	4	3	4	4	4	4	4	3
16	4	4	4	4	4	4	3	4	4	4	4	2	4	4
17	4	4	3	3	4	3	3	3	4	4	4	3	3	3
18	4	4	4	4	4	4	4	4	4	4	4	4	4	4
19	4	3	4	3	4	4	4	4	4	4	3	2	4	4
20	4	4	4	4	4	4	4	3	4	4	4	4	4	3
M*	**3.9**	**3.7**	**3.5**	**3.7**	**3.7**	**3.9**	**3.7**	**3.6**	**3.8**	**4**	**3.8**	**3.2**	**3.6**	**3.5**
*%***	**100%**	**95%**	**90%**	**95%**	**95%**	**100%**	**95%**	**100%**	**100%**	**100%**	**100%**	**80%**	**90%**	**95%**

Mean Degree of agreement (%) = Number of participants who gave the item a score of 3 or 4/Total number of participants (20) x100%**

Supplementary Material S2 Scores from the pilot test with 20 postpartum women, means scores and degree of agreement of each item of the scale
