# Supplementary material for: Cultural Adaptation, Validation and Evaluation of the Psychometric Properties of an Obstetric Violence Scale in the Spanish Context
Source: Nurs Rep. 2023 Oct 3;13(4):1368–87. doi: 10.3390/nursrep13040115 (PMC10594477; doi:10.3390/nursrep13040115)
Supplement: Supplementary file 1 [file nursrep-13-00115-s001.zip › nursrep-2604378-supplementary/Supplementary Material S3 Nursing Reports VO.pdf]

**Obstetric Violence Scale items**	*It does not describe what happened to me at all N (%)*	*I'm not sure but I believe/feel that this did not happen to me N (%)*	*I'm not sure N (%)*	*I'm not sure but I think/feel that this did happen to me N (%)*	*This definitely happened to me N (%)*
1- Members of the healthcare staff made ironic or derogative comments or made jokes about your behavior.	**236 (92.2%)**	**5 (2.0%)**	**3 (1.2%)**	**2 (0.8%)**	**10 (3.9%)**
2- You were addressed to with nicknames or diminutives (e.g., mummy, chubby, etc.) or treated as if you were unable to understand the processes you were going through.	**244 (95.3%)**	**6 (2.3%)**	**0 (0%)**	**3 (1.2%)**	**3 (1.2%)**
3- You felt treated as a child or neglected by the staff, as if you were unable to make decisions about what was happening to you before, during or after delivery.	**226 (88.3%)**	**12 (4.7%)**	**8 (3.1%)**	**2 (0.8%)**	**8 (3.1%)**
4- You were somehow criticized for expressing your emotions (cry, scream of pain, etc.) during labor or delivery.	**233 (91.0%)**	**6 (2.3%)**	**5 (2.0%)**	**2 (0.8%)**	**10 (3.9%)**
5- It was impossible for you to ask queries or express your fears or concerns because nobody answered, or they answered in a bad way.	**236 (92.2%)**	**8 (3.1%)**	**3 (1.2%)**	**2 (0.8%)**	**7 (2.7%)**
6-You were subjected to medical procedures without asking your consent or without explaining why such procedures were needed.	**217 (84.8%)**	**14 (5.5%)**	**6 (2.3%)**	**5 (2.0%)**	**14 (5.5%)**
7-At the moment of delivery, you were compelled to keep lying on your back despite you expressed you discomfort with that position.	**229 (89.5%)**	**7 (2.7%)**	**5 (2.0%)**	**6 (2.3%)**	**9 (3.5%)**
8-You were compelled to stay in bed and prevented from walking or seeking the position you needed.	**233 (91.0%)**	**8 (3.1%)**	**4 (1.6%)**	**4 (1.6%)**	**7 (2.7%)**
9-You were not allowed to be accompanied by someone you trusted in.	**243 (94.9%)**	**2 (0.8%)**	**2 (0.8%)**	**3 (1.2%)**	**6 (2.3%)**
10-You were prevented from having immediate contact with your	**237 (92.6%)**	**3 (1.2%)**	**3 (1.2%)**	**5 (2.0%)**	**8 (3.1%)**

newborn, before the doctor took him/her away (caressing, holding him/her in your arms, etc.).					
11-After delivery, they make you feel you had not behaved up to what was expected of you (that you had not "helped").	**247 (96.5%)**	**3 (1.2%)**	**2 (0.8%)**	**2 (0.8%)**	**2 (0.8%)**
12-Your childbirth care experience made you feel vulnerable, guilty or insecure in any sense.	**230 (89.8%)**	**8 (3.1%)**	**7 (2.7%)**	**5 (2.0%)**	**6 (2.3%)**
13-After delivery, you were denied the opportunity to use a birth control device or procedure (IUD, tubal ligation, etc.).	**246 (96.1%)**	**3 (1.2%)**	**3 (1.2%)**	**2 (0.8%)**	**2 (0.8%)**
14-During or after labor, you felt exposed to the gaze of other people unknown to you (exposure to strangers).	**212 (82.8%)**	**14 (5.5%)**	**4 (1.6%)**	**7 (2.7%)**	**19 (7.4%)**
N=Frequency %=Percentage					

Supplementary Material Table S3. Frequencies and response percentages of each item of Obstetric Violence Scale
