# Supplementary material for: Cultural Adaptation, Validation and Evaluation of the Psychometric Properties of an Obstetric Violence Scale in the Spanish Context
Source: Nurs Rep. 2023 Oct 3;13(4):1368–87. doi: 10.3390/nursrep13040115 (PMC10594477; doi:10.3390/nursrep13040115)
Supplement: Supplementary file 1 [file nursrep-13-00115-s001.zip › nursrep-2604378-supplementary/Supplementary Material S4 Nursing Reports VO.pdf]

FINAL JMLE ITEM STATISTICS

Item	Difficulty	Std. Error	WMS	Std. WMS	UMS	Std. UMS
v1	-0,09	0,09	1,09	0,47	1,17	0,49
v2			DROPPED			
v3	-0,08	0,10	0,85	-0,77	0,83	-0,50
v4	-0,11	0,09	1,18	0,92	1,38	1,00
v5	0,02	0,11	0,77	-0,99	0,58	-0,98
v6	-0,32	0,08	1,08	0,54	1,06	0,30
v7	-0,11	0,09	1,06	0,40	1,07	0,33
v8	-0,00	0,10	0,92	-0,30	0,76	-0,55
v9	0,10	0,11	1,16	0,69	1,16	0,45
v10	-0,03	0,10	1,02	0,18	0,95	0,05
v11	0,54	0,15	0,86	-0,27	0,37	-1,10
v12	0,02	0,10	0,58	-2,51	0,45	-2,10
v13	0,53	0,15	1,09	0,38	1,13	0,41
v14	-0,46	0,08	1,21	1,43	1,17	0,76

Item dropped due to unobserved categories. Collapse categories to retain item.

Supplementary Material Table S4. First Rasch analysis by Joint Maximum Likelihood estimation (JMLE) with all the items.
