# Supplementary material for: Cultural Adaptation, Validation and Evaluation of the Psychometric Properties of an Obstetric Violence Scale in the Spanish Context
Source: Nurs Rep. 2023 Oct 3;13(4):1368–87. doi: 10.3390/nursrep13040115 (PMC10594477; doi:10.3390/nursrep13040115)
Supplement: Supplementary file 1 [file nursrep-13-00115-s001.zip › nursrep-2604378-supplementary/Supplementary Material S5 Nursing Reports VO.pdf]

CORRELATION MATRIX

	v1	v3	v4	v5	v6	v7	v8	v9	v10	v11	v12	v13	v14
v1	1,0000	0,1957	0,3350	0,0299	-0,1942	-0,0632	-0,3149	-0,1424	-0,2318	-0,2053	-0,1070	-0,0744	-0,3118
v3	0,1957	1,0000	-0,0162	0,2082	-0,0100	-0,2608	-0,2807	-0,0955	-0,1565	-0,0448	0,2528	-0,2464	-0,3248
v4	0,3350	-0,0162	1,0000	-0,0261	-0,2984	-0,1485	-0,2952	-0,1721	-0,1718	0,0330	-0,0297	-0,0631	-0,1926
v5	0,0299	0,2082	-0,0261	1,0000	-0,0823	-0,2700	-0,1982	-0,2780	-0,0809	-0,0720	0,2525	-0,2074	-0,0542
v6	-0,1942	-0,0100	-0,2984	-0,0823	1,0000	-0,2574	-0,1494	-0,0508	0,0335	-0,0453	-0,0764	-0,0475	-0,0105
v7	-0,0632	-0,2608	-0,1485	-0,2700	-0,2574	1,0000	0,5224	0,0141	-0,2068	-0,1065	-0,1750	0,0241	-0,1515
v8	-0,3149	-0,2807	-0,2952	-0,1982	-0,1494	0,5224	1,0000	-0,0166	-0,0281	-0,1878	-0,1521	0,1069	-0,0309
v9	-0,1424	-0,0955	-0,1721	-0,2780	-0,0508	0,0141	-0,0166	1,0000	0,1919	-0,2063	-0,1278	0,0670	-0,2004
v10	-0,2318	-0,1565	-0,1718	-0,0809	0,0335	-0,2068	-0,0281	0,1919	1,0000	-0,1772	-0,2613	-0,0000	-0,0359
v11	-0,2053	-0,0448	0,0330	-0,0720	-0,0453	-0,1065	-0,1878	-0,2063	-0,1772	1,0000	0,2767	0,0257	0,1483
v12	-0,1070	0,2528	-0,0297	0,2525	-0,0764	-0,1750	-0,1521	-0,1278	-0,2613	0,2767	1,0000	-0,3090	-0,1139
v13	-0,0744	-0,2464	-0,0631	-0,2074	-0,0475	0,0241	0,1069	0,0670	-0,0000	0,0257	-0,3090	1,0000	-0,0406
v14	-0,3118	-0,3248	-0,1926	-0,0542	-0,0105	-0,1515	-0,0309	-0,2004	-0,0359	0,1483	-0,1139	-0,0406	1,0000

Supplementary Material S5 Correlation matrix. Yen Q3 Test
