# Supplementary material for: Cultural Adaptation, Validation and Evaluation of the Psychometric Properties of an Obstetric Violence Scale in the Spanish Context
Source: Nurs Rep. 2023 Oct 3;13(4):1368–87. doi: 10.3390/nursrep13040115 (PMC10594477; doi:10.3390/nursrep13040115)
Supplement: Supplementary file 1 [file nursrep-13-00115-s001.zip › nursrep-2604378-supplementary/Supplementary Material S7 Nuersing Reports VO.pdf]

Primary School (n=61) (a)	Secondary School (n=103) (b)	University (n=92) (c)			Kruskall Wallis Test	Effect size	Post Hoc Test
M(SD)	M(SD)	M(SD)		X^2	Value p	$Ɛ^2$	Dwass-Steel-Critchlow-Fligne
1.48 (3.49)	3.06 (5.83)	4.03 (8.45)		5.24	0.073	0.002	No differences between groups
**Normal vaginal delivery (n=199) (a)**	**Dystocic vaginal delivery with forceps (n=17) (b)**	**Dystocic delivery with vacuum (n=7) (c)**	**Unplanned cesarean section (n=33) (d)**		**Kruskall Wallis Test**	**Effect size**	**Post Hoc Test**
M(SD)	M(SD)	M(SD)	M(SD)	X^2	Value p	$Ɛ^2$	Dwass-Steel-Critchlow-Fligne
2.01 (4.50)	6.35 (9.57)	1.86 (4.49)	7.76 (11.45)	15.06	0.001*	0.006	a,d
**Center 1 (n=188) (a)**	**Center 2 (n=24) (b)**	**Center 3 (n=29) ©**	**Center 4 (n=15) (d)**		**Kruskall Wallis Test**	**Effect size**	**Post Hoc Test**
M(SD)	M(SD)	M(SD)	M(SD)	X^2	Value p	$Ɛ^2$	Dwass-Steel-Critchlow-Fligne
3.44 (6.98)	1.17 (2.96)	3.00 (7.04)	0.93 (1.58)	4.23	0.238	0.016	No differences between groups

M(SD): Mean(Standard Deviation).
X^2=Chi Squared
$Ɛ^2$= Epsilon squared. Effect size from 0 to 1 (1 being maximum effect).

Supplementary Material Table S7. Means, standard deviations, p-value, contrast statistic and effect size for variables in more than two groups.
