# Supplementary material for: Cultural Adaptation, Validation and Evaluation of the Psychometric Properties of an Obstetric Violence Scale in the Spanish Context
Source: Nurs Rep. 2023 Oct 3;13(4):1368–87. doi: 10.3390/nursrep13040115 (PMC10594477; doi:10.3390/nursrep13040115)
Supplement: Supplementary file 1 [file nursrep-13-00115-s001.zip › nursrep-2604378-supplementary/Supplementary Material Table S1 Nursing Reports VO.pdf]

	PROFESSION	GENDER	YEARS OF EXPERIENCE	PROFILE AND REASONS TO CHOOSE
**Expert 1**	Midwife	Woman	25	Coordinator of the Low Intervention Childbirth group of the Andalusian Association of Midwives. Lecturer in Perinatal Mental Health training. Choice: Women's rights activist in childbirth
**Expert 2**	Nurse	Woman	15	PhD. Associate Professor at the Universitat Jaume I (Castelló). Collaborator in nursing degree studies and with the advanced breastfeeding program at Blanquerna University. Published multiple studies on Obstetric Violence and creator of the PercOV-S questionnaire. Choice: In our opinion, the leading expert on obstetrical violence in our country. Creator of the PercOV-S questionnaire.
**Expert 3**	Midwife	Woman	30	Midwife in Primary Care in Gran Canaria. Teaching collaborator. Responsible for the area of midwifery in the College of Nursing of Las Palmas for 20 years. Choice: After her long professional experience, she knows the testimony of thousands of women.
**Expert 4**	Midwife	Woman	20	PhD. Part-time lecturer at the University of La Laguna and works in assistance programs in the Canary Health Service over the area of women. She is a member of the group "Obstetric Violence Observatory" of the Ministry of Health. Choice: Knows the legal institutional environment of obstetric violence as a member of the group Obstetric Violence Observatory of the Ministry of Health
**Expert 5**	Obstetrician	Man	35	PhD. Part-time Lecturer at the University of Las Palmas de Gran Canaria. Head of Obstetrics and Gynecology Section of the Complejo Hospitalario Universitario Insular Materno Infantil de Canarias. Master in Bioethics. Member of the Provincial Ethics Committee. Choice: Expert in bioethics and patients' rights, in addition to being an obstetrician with a long professional career.
**Expert 6**	Midwife	Woman	20	Midwife attached to the Multiprofessional Teaching Unit of Obstetrics and Gynecology. Speaker at national and international congresses and conferences related to maternity, childbirth, lactation and pelvic floor. Author of research papers published in national and international scientific journals. IP research study in collaboration with the Pelvic Floor Rehabilitation Unit of the Negrín Hospital. Choice: Responsible for the training of midwives in the study environment, in addition to training as a midwife in a country (UK) where there is a different sensitivity in childbirth care.

**Expert 7**	Midwife	Woman	10	Midwife at Complejo Hospitalario Universitario Insular Materno Infantil de Canarias. Teaching collaborator, assistance and TFE tutor in the Multidisciplinary Unit of Obstetrics and Gynecology. President of ACAMAT (Canarian Association of Midwifes). Doctoral thesis on Obstetric Violence. Choice: She is doing a doctoral thesis on obstetric violence. She has a deep knowledge of the theoretical framework.
**Expert 8**	Obstetrician	Woman	8	Gynecologist and Obstetrician at the General Hospital of Fuerteventura Virgen de la Peña. Choice: Having worked in two of the hospitals where the study has been carried out (HUMIC-Gran Canaria and Fuerteventura), she can provide a double vision, as they are two very different obstetric care contexts.

Supplementary Material Table S1. Professional profiles of the experts participating in the content validation process
