# Supplementary material for: Cultural Adaptation, Validation and Evaluation of the Psychometric Properties of an Obstetric Violence Scale in the Spanish Context
Source: Nurs Rep. 2023 Oct 3;13(4):1368–87. doi: 10.3390/nursrep13040115 (PMC10594477; doi:10.3390/nursrep13040115)
Supplement: Supplementary file 1 [file nursrep-13-00115-s001.zip › nursrep-2604378-supplementary/Supplementary Material Table S6 Nursing Reports VO.pdf]

ESCALA DE VIOLENCIA OBSTÉTRICA

A continuación, se le muestran una serie de preguntas sobre aspectos que se han relacionado con la violencia obstétrica hacia las mujeres. Nos gustaría saber si en su último parto, según sus recuerdos, usted vivió alguna de estas situaciones.

Puntúe la escala según la siguiente guía:

0- No describe para nada lo que me ocurrió

1- No estoy segura pero creo/siento que no me ocurrió

2- No estoy segura

3- No estoy segura pero creo/siento que si me ocurrió

4- Definitivamente esto me ocurrió

ÍTEM 1: El personal de salud hizo comentarios irónicos, descalificadores o en tono de chiste acerca de tu comportamiento.	0 1 2 3 4
ÍTEM 3: Te sentiste infantilizada o anulada por el equipo médico como si fueras incapaz de tomar decisiones sobre lo que te ocurría antes, durante o después del parto.	0 1 2 3 4
ÍTEM 4: Fuiste criticada de algún modo por expresar tus emociones (como llorar, gritar de dolor, etc) durante el trabajo de parto y/o el parto.	0 1 2 3 4
ÍTEM 5: Te fue difícil o imposible preguntar o manifestar tus miedos o inquietudes porque no te respondían o lo hacían de mala manera.	0 1 2 3 4
ÍTEM 6: Te realizaron procedimientos médicos sin pedirte consentimiento sin explicarte por qué eran necesarios.	0 1 2 3 4

ÍTEM 7: En el momento del parto, te obligaron a permanecer acostada boca arriba aunque manifestaras tu incomodidad en esta posición.	0 1 2 3 4
ÍTEM 8: Fuiste obligada a quedarte en cama impidiéndote caminar o buscar posiciones según tus necesidades.	0 1 2 3 4
ÍTEM 9: Se te impidió estar acompañada por alguien de tu confianza	0 1 2 3 4
ÍTEM 10: Se te impidió el contacto inmediato con tu hija/o recién nacida/o antes de que se lo llevara la/el neonatólogo para control (acariciarlo, tenerlo en brazos, etc.).	0 1 2 3 4
ÍTEM 11: Después del parto, te hicieron sentir que no habías estado a la altura de lo que se esperaba de ti ("que no habías «colaborado").	0 1 2 3 4
ÍTEM 12: La experiencia de la atención en el parto te hizo sentir vulnerable, culpable o insegura en algún sentido.	0 1 2 3 4
ÍTEM 13: Después del parto se te negó la posibilidad de utilizar algún dispositivo o realizar algún procedimiento de control de la natalidad (DIU, ligadura de trompas..etc).	0 1 2 3 4
ÍTEM 14: Durante el trabajo del parto o con posterioridad a este te sentiste expuesta a la mirada de otras personas desconocidas para ti (exposición a extraños).	0 1 2 3 4

Puntuación de Violencia Obstétrica: Sumar las puntuaciones obtenidas en cada ítem Máximo 52 puntos-Mínimo 0 puntos.
